# Long-term prognostic factors for PRRT in neuroendocrine tumors

**DOI:** 10.3389/fmed.2023.1169970

**Published:** 2023-06-09

**Authors:** Nils Florian Trautwein, Johannes Schwenck, Johann Jacoby, Gerald Reischl, Francesco Fiz, Lars Zender, Helmut Dittmann, Martina Hinterleitner, Christian la Fougère

**Affiliations:** ^1^Department of Nuclear Medicine and Clinical Molecular Imaging, University Hospital of Tübingen, Tübingen, Germany; ^2^Werner Siemens Imaging Center, Department of Preclinical Imaging and Radiopharmacy, Eberhard Karls University, Tübingen, Germany; ^3^ENETS Center of Excellence, University Hospital of Tübingen, Tübingen, Germany; ^4^Cluster of Excellence iFIT (EXC 2180) “Image-Guided and Functionally Instructed Tumor Therapies”, Eberhard Karls University, Tübingen, Germany; ^5^Institute for Clinical Epidemiology and Applied Biometry, University Hospital of Tübingen, Tübingen, Germany; ^6^Department of Nuclear Medicine, E.O. Ospedali Galliera, Genoa, Italy; ^7^Department of Internal Medicine VIII, University Hospital of Tübingen, Tübingen, Germany; ^8^German Cancer Consortium (DKTK), German Cancer Research Center (DKFZ) Partner Site Tübingen, Tübingen, Germany

**Keywords:** neuroendocrine tumor (NET), ^177^Lu, DOTA-TATE, peptide receptor radionuclide therapy (PRRT), Ga-HA-DOTATATE, molecular tumor volume

## Abstract

**Aim/introduction:**

Peptide receptor radionuclide therapy (PRRT) is an effective and well-tolerated treatment option for patients with neuroendocrine tumors (NETs) that prolongs progression-free survival (PFS). However, the limited overall survival (OS) rates in the prospective phase III study (NETTER1) highlighted the need to identify patient-specific long-term prognostic markers to avoid unnecessary side effects and enable better treatment stratification. Therefore, we retrospectively analyzed prognostic risk factors in NET patients treated with PRRT.

**Methods:**

A total of 62 NET patients (G1: 33.9%, G2 62.9%, and G3 3.2%) with at least 2 cycles of PRRT with [^177^Lu]Lu-HA-DOTATATE (mean 4 cycles) were analyzed. Of which, 53 patients had primary tumors in the gastroenteropancreatic (GEP) system, 6 had bronchopulmonary NET, and 3 had NET of unknown origin. [^68^Ga]Ga-HA-DOTATATE PET/CT scans were performed before PRRT start and after the second treatment cycle. Different clinical laboratory parameters, as well as PET parameters, such as SUVmean, SUVmax, and PET-based molecular tumor volume (MTV), were collected, and their impact on the OS was investigated. Patient data with a mean follow-up of 62 months (range 20–105) were analyzed.

**Results:**

According to interim PET/CT, 16 patients (25.8%) presented with partial response (PR), 38 (61.2%) with stable disease (SD), and 7 (11.3%) with progressive disease (PD). The 5-year OS was 61.8% for all patients, while bronchopulmonary NETs showed poorer OS than GEP-NETs. Multivariable Cox regression analysis showed that chromogranin A level and MTV together were highly significant predictors of therapeutic outcome (HR 2.67; 95% CI 1.41–4.91; *p* = 0.002). Treatment response was also influenced by the LDH level (HR 0.98; 95% CI 0.9–1.0; *p* = 0.007) and patient age (HR 1.15; 95% CI 1.08–1.23; *p* < 0.001). ROC analysis revealed baseline MTV > 112.5 ml [Sens. 91%; Spec. 50%; AUC 0.67 (95% CI 0.51–0.84, *p* = 0.043)] and chromogranin A >1,250.75 μg/l [Sens. 87%; Spec. 56%; AUC 0.73 (95% CI 0.57–0.88, *p* = 0.009)] as the best cutoff values for identifying patients with worse 5-year survival.

**Conclusion:**

Our retrospective analysis defined MTV and chromogranin A in combination as significant prognostic factors for long-term OS. Furthermore, an interim PET/CT after two cycles has the potential in identifying non-responders who may benefit from a change in therapy at an early stage.

## Introduction

Neuroendocrine tumors (NETs) originate from the neuroendocrine system and can synthesize and secrete different neuro amines and peptides (1). Although still a fairly rare subtype of cancer, NETs have become more common over the past few decades (2, 3). Most NETs remain asymptomatic until they have spread. Therefore, more than 40% of NET patients have metastatic disease at the time of first diagnosis (4). For this reason, curative surgery is often no longer possible, and alternative treatments must be considered.

Somatostatin receptors (SSTR) are overexpressed by most well and moderately differentiated gastroenteropancreatic neuroendocrine tumors (GEP-NETs) by 80–100% (5) and are currently the most important target for treatment stratification. The interaction between the SSTR and somatostatin can lead to a profound treatment response, including the suppression of cell secretion and cell proliferation (6), and thus, the number of SSTR-targeting therapies for NETs has grown since the early 2000s. For GEP-NET G1 and G2, the prospective studies CLARINET and PROMID showed that antiproliferative somatostatin analog (SSA) therapies prolonged patients' progression-free survival compared to placebo (7, 8). Since the early 1990s, the combination of SSA coupled with a radioactive beta emitter (peptide receptor radionuclide therapy; PRRT) has been used as a treatment strategy for SSTR-positive NET (9), showing promising results, especially in patients suffering from GEP-NET (10). Moreover, the first multicenter prospective phase III clinical trial (NETTER1) revealed that patients treated with PRRT had a longer progression-free survival (PFS) when compared to high-dose SSA monotherapy. After 20 months of randomization, the rate of PFS was significantly higher in the PRRT group (65.2%) than in the control group (10.8%) (11). Recently, the long-term follow-up data were published, showing a difference of 11.7 months in median overall survival between the PRRT group (48 months) and controls (36.3 months), which did not achieve statistical significance (12). The current European Neuroendocrine Tumor Society (ENETS) guidelines recommend the use of PRRT as a second- to third-line therapy after progression under SSA in metastasized intestinal (midgut) NETs and as a third-line therapy in pancreatic NET with advanced locoregional disease (13). Data for PRRT in bronchopulmonary carcinoma are still rare. The comparatively low number of bronchopulmonary NETs that express enough somatostatin receptors to qualify as therapy candidates is an important issue (14). However, in a large retrospective study with over 100 patients and several smaller cohorts, PRRT was shown to be a well-tolerated treatment option for bronchopulmonary carcinoma (15, 16). These studies showed a PFS of 19–59 months; therefore, PRRT was included as a treatment option for bronchopulmonary carcinoma in the ESMO guidelines (17, 18).

Measuring sufficient SSTR expression by pre therapeutic PET or SPECT imaging is an important prerequisite for selecting patients for PRRT. Although adequate SSTR expression has been measured, insufficient response to PRRT can, however, occur at a rate that has been estimated between 15 and 30%; moreover, there are no established biomarkers for the prediction of long-term response and survival (19, 20).

Therefore, this retrospective analysis aimed to explore the prognostic value of different clinical parameters as biomarkers for long-term response to PRRT.

## Materials and methods

### Patients and PRRT

We screened our database for patients who received PRRT between February 2013 and February 2019 at the University Hospital of Tübingen. Only patients with tumor SSTR expression higher than the liver in a pre-therapeutic [^68^Ga]Ga-HA-DOTATATE-PET/CT scan were treated (21). PRRT was performed according to the practical guidelines of the Joint International Atomic Energy Agency (IAEA), the European Association of Nuclear Medicine (EANM), and the Society of Nuclear Medicine and Molecular Imaging (SNMMI), in accordance with the Rotterdam Protocol (22).

Patients were treated for a median of four cycles, each including an intravenous administration of 7,180 ± 650 MBq [^177^Lu]Lu-HA-DOTATATE per cycle which was accompanied by an amino acid solution for renal protection (23). Patients were treated with at least two and a maximum of nine cycles (Table 1). The median time between the two cycles was 14 weeks (range: 8–24 weeks). The goal was to administer four cycles of PRRT; in some patients, the number of cycles was not achieved due to individual circumstances. In the case of more than four cycles per patient, retreatments were performed. In 18 patients, salvage PRRT was carried out during the follow-up period. In five patients, the administered activity was reduced because of impaired renal function or other relevant secondary diseases. SSA therapy was maintained during PRRT; however, a time interval of at least 4 weeks between the last SSA administration and PRRT was ensured. No other oncological treatments were performed in addition to SSA therapy, but supportive therapies, such as antidiarrheal medications or antibiotics, were administered to patients according to their individual needs.

**Table 1:** Patients' characteristics.

**Number of patients**	**62**
Age, median (range) in years	64 (27–80)
**Gender**, ***n*** **(%)**	
Male	36 (58%)
Female	26 (42%)
**Prior therapies**, ***n*** **(%)**	
Surgery	49 (79%)
Somatostatin analog	44 (71%)
Systemic therapies	6 (10%)
PRRT	9 (15%)
Local therapies (SIRT, RFA, and TACE)	4 (6%)
**Primary tumor site**, ***n*** **(%) and grading**, ***n*** **(%)**	
Gastroenteropancreatic	53 (85%)
G1	20 (38%)
G2	31 (58%)
G3	2 (4%)
Bronchopulmonary	6 (10%)
G1	0 (0%)
G2	6 (100%)
G3	0 (0%)
Cancer of unknown primary	3 (5%)
G1	1 (33%)
G2	2 (67%)
G3	0 (0%)
**Cycles of PRRT**, ***n*** **(%)**	
2	7 (11%)
3	10 (16%)
4	31 (50%)
>4	14 (22%)

SIRT, selective internal radiation therapy; RFA, radiofrequency ablation; TACE, trans-arterial chemoembolization; G, grading.

[^177^Lu]Lu-HA-DOTATATE was prepared according to good manufacturing practice and the German Medicinal Products Act (AMG § 13 2b). Interim [^68^Ga]Ga-HA-DOTATATE-PET/CT scans were performed after two cycles of PRRT. Blood counts and creatinine were monitored on the day of [^177^Lu]Lu-HA-DOTATATE therapy injection. Side effects were monitored according to the Common Terminology Criteria for Adverse Events (CTCAE v5.0) (24).

### PET/CT image acquisition

A baseline [^68^Ga]Ga-HA-DOTATATE-PET/CT scan was performed, on average, a median of 7 (range: 0–17) weeks before PRRT. A median of 11 (range: 6–32) weeks after the second cycle, but before the administration of the third cycle, a PET scan was conducted for imaging. All scans were conducted on a state-of-the-art PET/CT scanner (Biograph mCT, Siemens Healthineers) 45 min p.i. after i.v. injection of 2 MBq kg/BW [^68^Ga]Ga-HA-DOTATATE (25). Additionally, a diagnostic CT scan including contrast enhancement in arterial and portal venous phase (120 ml of Ultravist 370b^®^, Bayer Healthcare Pharmaceuticals; flow rate 2.5 ml/s) was performed. In patients with contraindications to contrast agents, a diagnostic CT scan without a contrast medium was performed. Data were corrected for attenuation as well as scattered and reconstructed with OSEM3D including time of flight and point spread functions (2 iterations, 21 subsets, and Gaussian filter 2 mm).

### PET/CT image interpretation

The [^68^Ga]Ga-HA-DOTATATE PET-based assessment of the SSTR molecular tumor volume (MTV) was performed by semi-automatic volumetric segmentation of non-physiologic tracer uptake using the software tool Affinity Hybrid Viewer (Hermes Medical Solution, Sweden).

Pathologic SSTR expression was defined as standardized uptake values (SUV), which were higher than the 1.5-fold mean SUV of the liver plus two times the standard deviation (SD).


(1) $$\begin{array}{l} MTV=SUV_{tumor}>{1.5\;\times \;SUVmean}_{liver}\;+2\;\times SD_{liver} \end{array}$$

SUVmean, SUVmax, and SD of the liver were determined by a 5 ml spherical volume of interest (VOI) in the left liver lobe. A 3 ml spherical VOI in the fifth lumbar vertebrae was used to assess the SUV parameters of the bones, whereas a 5 ml spherical VOI was used to measure the SUV characteristics of the spleen. First, the “single click segmentation” tool was used to mark all regions with an SUV value higher than the reference SUV. Furthermore, several volumes of interest (VOIs) were formed. Then, the semi-automatically segmented areas were selected and reviewed by a trained physician, who excluded physiological SSTR-expressing areas (e.g., the kidney and pituitary gland) and non-disease-related lesions (Supplementary Figure 1).

SUVmax and MTV-based SUVmean of the largest metastatic tumor lesion were evaluated. The SUV was calculated based on body weight.

Early therapy response was estimated by the relative change in MTV after the first two PRRT cycles. As described by Ohlendorf et al., partial response (PR) was defined as a reduction in MTV of more than 73% and progressive disease (PD) as an increase in MTV of more than 63%, whereas stable disease (SD) was defined between the two upper values (26). PR and SD were considered as responders, a non-responder was defined by PD. For one patient, a PET/CT after the second PRRT cycle was not available. Furthermore, response to treatment after two cycles of PRRT was assessed using CT or MR images according to RECIST 1.1 (27).

### Statistical analysis

For the whole statistical analysis, patient data with a follow-up for a maximum of 8 years were analyzed. The SUV parameters of responders and non-responders were not normally distributed and were therefore compared with Mann–Whitney *U*-test. With the use of univariable and multivariable Cox regression, the prognostic value of different variables was assessed. Due to the skewed distribution of the γ-GT, the MTV, and the chromogranin A in our cohort, the values were log-transformed. The MTV is an image morphological biomarker, and chromogranin A is a blood-based biomarker for the tumor burden of the patients. For the predictors, MTV and chromogranin A alone, the assumption of proportional hazards is not plausible. To solve this problem and due to the strong correlation between MTV and chromogranin A, these two factors were combined. For this purpose, the values were first z-standardized and then averaged. The assumption of constant hazards ratio (HR) was examined using the derived Schoenfeld residuals.

To identify optimal cutoff values for 5 years OS, a receiver operating characteristic (ROC) analysis was performed. The OS was determined as time in months from the baseline PET/CT to death from any cause. The OS was evaluated using the Kaplan–Meier technique. Two distinct groups were compared using a log-rank test. The statistical analysis was performed using GraphPad Prism 9.4 and R version 4.1.1.

The institutional ethics committee of our institution approved this retrospective analysis (Decision 530/220BO). Due to the retrospective nature of this study, the requirement to obtain informed consent was waived.

## Results

### Patients

**Figure 1:**
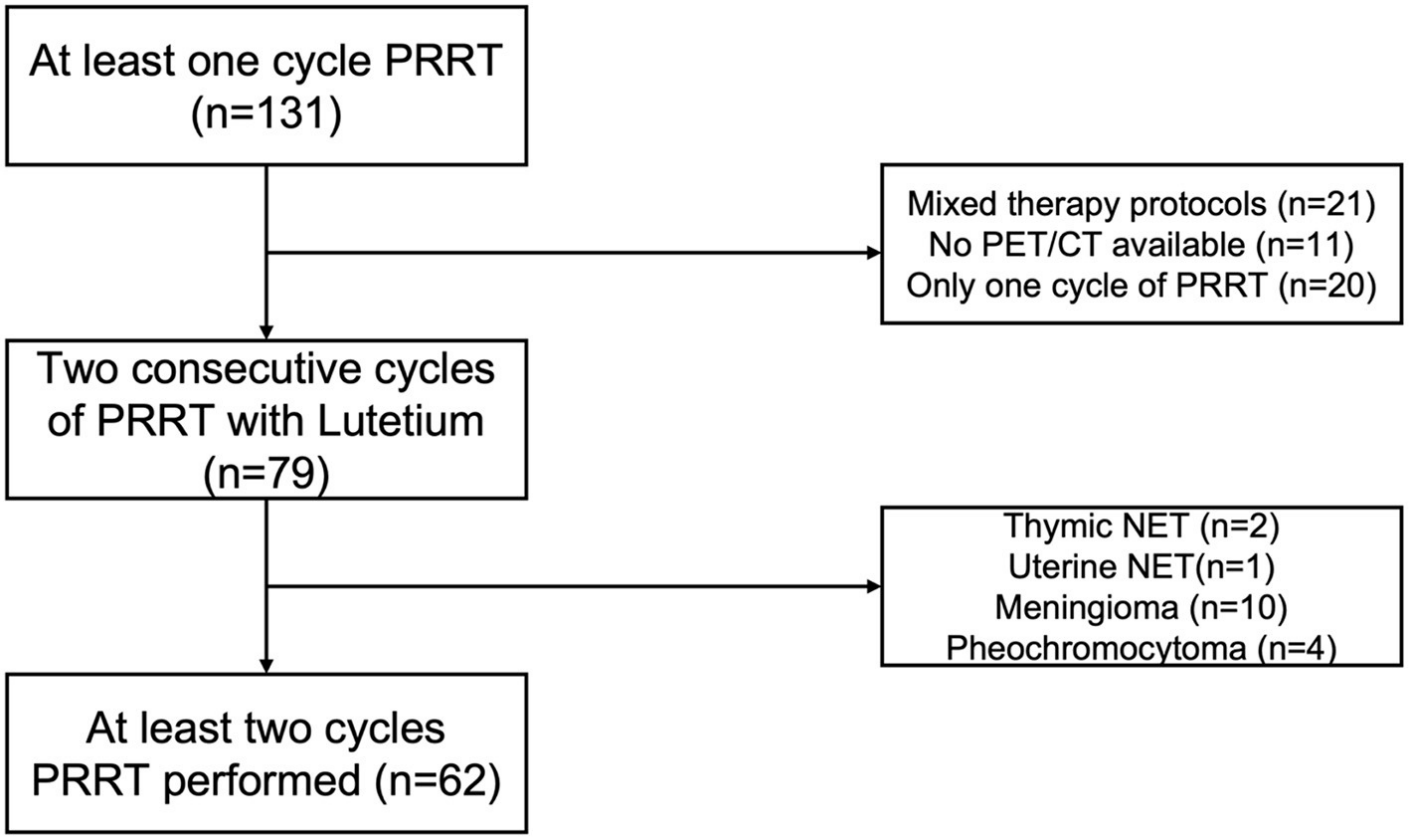
Patient selection algorithm.

Between February 2013 and February 2019, 131 patients were treated with PRRT in our department. First, we excluded all patients with only one cycle of PRRT (*n* = 20), mixed protocols including [^90^Y] Yttrium (*n* = 21), or no available baseline PET/CT (*n* = 11). Then, patients were selected according to their histological classifications, excluding meningiomas (*n* = 10), pheochromocytomas (*n* = 4), thymic (*n* = 1), or uterine (*n* = 1) NETs (Figure 1). The remaining 62 patients had a histologically confirmed gastroenteropancreatic, bronchopulmonary, or CUP-NET and were treated with a median of four cycles (range 2–9), but at least with two consecutive cycles of [^177^Lu]Lu-HA-DOTATATE. The mean of the administered cumulative activity of all cycles per patient was 29,665 MBq (range: 11,503–59,446 MBq). The median time between the two cycles was 14 weeks (range: 8–24 weeks). The median follow-up time was 62 months (range 33–104 months). To assess whether the longer treatment interval might have an impact on our data, an additional analysis was performed in which patients with a longer interval than the mean and two standard deviations between two treatment cycles were excluded (Supplementary Figure 1).

Patients' characteristics are summarized in Table 1. In total, 21 patients (33.9%) suffered from WHO grade 1 (G1) NET, 39 from grade 2 (G2) NET (62.9%), and 2 (3.2%) from grade 3 (G3) NET. The majority of the patients (*n* = 53) were diagnosed with gastroenteropancreatic (GEP)-NET (85.4%), six had bronchopulmonary NET (9.7%), and three had CUP-NET (4.8%). One CUP-NET patient probably had a GEP-NET histologically. For the others, no inference of the primary region could be obtained by histology. Baseline and interim PET parameters are presented in Tables 2, 3. Treatment-related adverse events according to CTCAE v5.0 are displayed in Table 4. One patient developed a myelodysplastic syndrome (MDS) during the follow-up period.

**Table 2:** PET parameters of the baseline PET scan.

**PET parameters**	**All (*n =* 62) Mean (SD)**	**PR (*n =* 16) Mean (SD)**	**SD (*n =* 38) Mean (SD)**	**PD (*n = 7*) Mean (SD)**
SUV_mean_ liver	4.41 (1.14)	4.30 (1.23)	4.58 (1.18)	4.10 (0.53)
SUV_max_ liver	6.28 (1.53)	6.03 (1.61)	6.62 (1.60)	5.81 (0.62)
SUV_mean_ spleen	14.57 (5.13)	14.21 (4.15)	14.92 (5.64)	14.22 (5.72)
SUV_max_ spleen	17.86 (6.22)	17.20 (4.90)	18.25 (6.91)	17.91 (6.88)
SUV_mean_ bone	0.97 (0.39)	1.01 (0.40)	0.94 (0.41)	0.93 (0.36)
SUV_max_ bone	1.82 (1.12)	2.00 (1.56)	1.69 (0.68)	1.74 (0.69)
SUV_mean_ tumor	12.21 (3.97)	12.52 (4.99)	12.51 (3.26)	10.21 (1.92)
SUV_max_ tumor	25.87 (14.14)	28.61 (18.06)	25.67 (10.71)	17.98 (7.38)
MTV in ml	121.5 (238.1)	69.54 (35.26)	168.1 (125.7)	114.6 (182.9)

SUV values did not differ significantly (*p* > 0.05).

**Table 3:** PET parameters of the interim PET scan after two cycles of PRRT.

**PET parameters**	**All (*n =* 62) Mean (SD)**	**PR (*n =* 16) Mean (SD)**	**SD (*n =* 38) Mean (SD)**	**PD (*n =* 7) Mean (SD)**
SUV_mean_ liver	4.92 (1.30)	4.92 (1.16)	5.06 (1.41)	4.31 (1.28)
SUV_max_ liver	6.94 (1.89)	6.64 (1.41)	7.37 (2.19)	6.23 (1.92)
SUV_mean_ spleen	15.87 (5.46)	15.21 (4.37)	17.09 (6.19)	13.90 (5.19)
SUV_max_ spleen	19.59 (6.53)	18.42 (4.95)	21.07 (7.46)	17.95 (6.61)
SUV_mean_ bone	0.93 (0.27)	0.88 (0.22)	0.99 (0.31)	0.84 (0.23)
SUV_max_ bone	1.72 (0.55)	1.66 (0.60)	1.77 (0.52)	1.71 (0.54)
SUV_mean_ tumor	13.10 (4.18)	12.83 (4.18)	13.93 (4.29)	10.53 (2.86)
SUV_max_ tumor	26.48 (13.22)	24.49 (14.70)	30.25 (12.44)	17.12 (3.28)
MTV in ml	112.3 (250.1)	23.36 (33.95)	157.1 (306.9)	227.9 (335.3)

SUV values did not differ significantly (*p* > 0.05).

**Table 4:** Adverse Events according to CTCAE v5.0.

**Events**	**Grade 1**	**Grade 2**	**Grade 3**	**Grade 4**
Anemia	7	0	0	0
Platelets	17	1	1	0
White blood cells	6	1	1	0

CTCAE, Common terminology criteria for adverse events.

### Clinical imaging and response assessment

**Figure 2:**
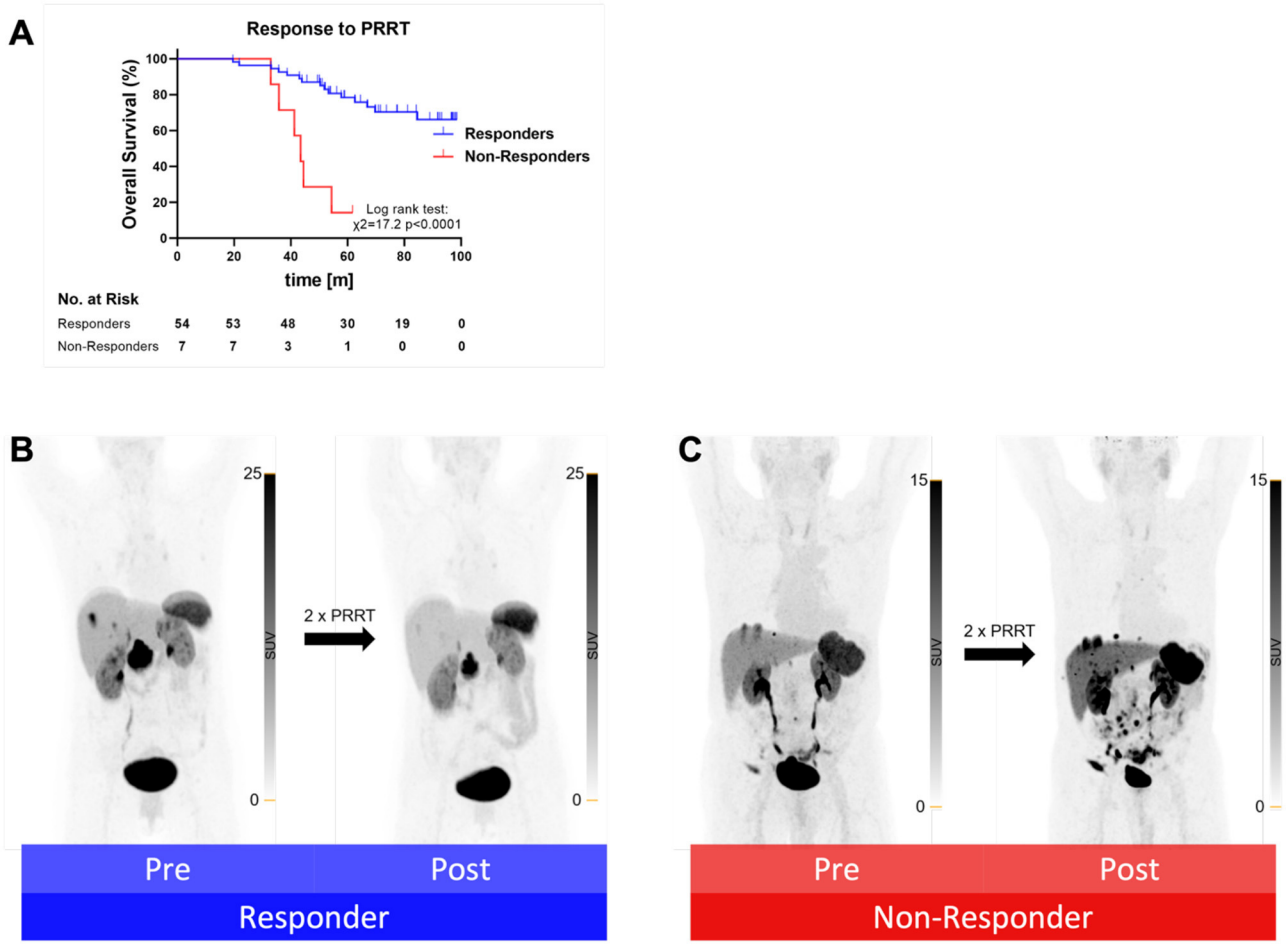
**(A)** Kaplan–Meier curves for overall survival separated by responders (blue) to PRRT after two cycles and non-responders (red) after two cycles. m, months. Exemplary the maximum intensity projection (MIP) of a PET from a responder **(B)** and a non-responder **(C)** pre and post two cycles of PRRT.

According to the clinical follow-up PET/CT scan after the second PRRT cycle, 16 out of 62 patients (25.8%) presented with a PR, while an SD was observed in 38 patients (61.2%). Seven patients (11.3%) suffered from PD and were defined as non-responders. Non-responders displayed a significantly worse OS than responders (Figure 2). Baseline SUV values from responders and non-responders did not differ significantly (*p* > 0.05). Furthermore, the response to PRRT after two cycles were assessed according to RECIST 1.1. In total, 44 patients showed an SD, 11 patients showed a PR, and 6 patients suffered from a PD (Table 5). No CR was achieved in any patient. For one patient, a PET/CT after the second PRRT cycle was not available.

**Table 5:** Treatment response to two cycles of PRRT, according to RECIST 1.1 and MTV-based.

**RECIST 1.1**	**CR**	**PR**	**SD**	**PD**
Patients *n*, (%)	0 (0)	11 (18)	44 (72)	6 (10)
**MTV-based**				
Patients *n*, (%)		16 (26)	38 (62)	7 (12)

### Prognostic factors for overall survival

**Figure 3:**
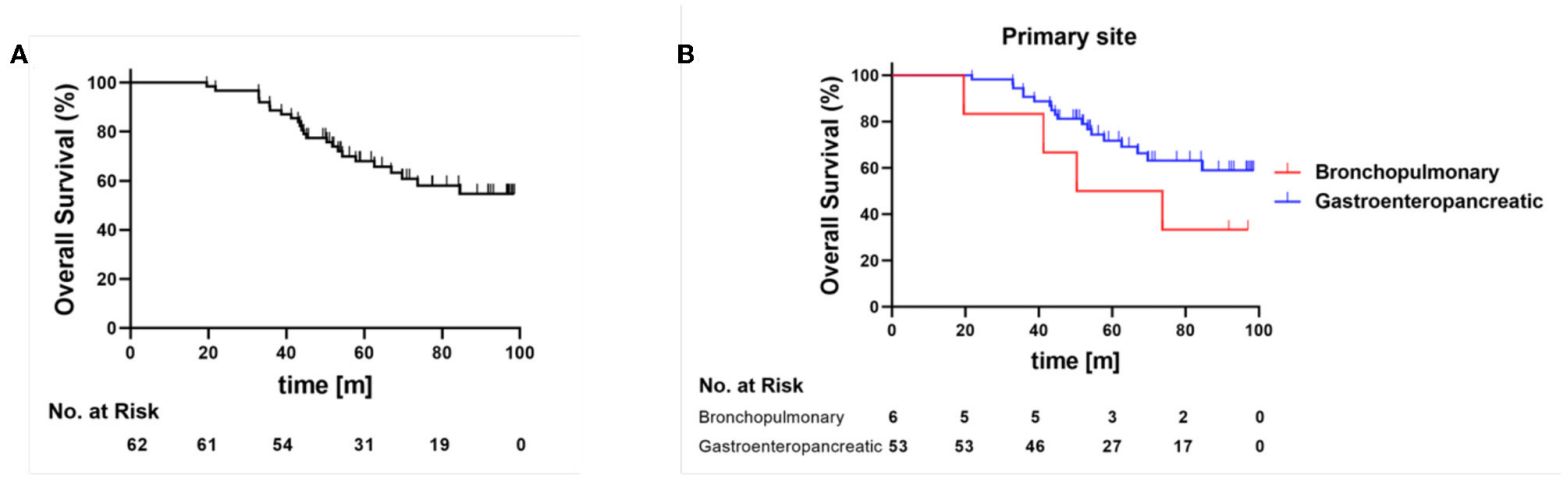
Overall survival for all patients **(A)** and in dependence of primary side **(B)**, Bronchopulmonary NET red line, gastroenteropancreatic NET blue line, m, months.

The 5-year OS was 61.8% for all patients, while bronchopulmonary NETs (5-year OS 50%) showed a poorer OS than GEP-NETs (5-year OS 69.5%) (Figure 3). In the univariable Cox regression, a combination of MTV, derived from the baseline [^68^Ga]Ga-HA-DOTATATE PET, and chromogranin A (*p* < 0.001) were risk factors and correlated with a significantly lower probability of survival. The γ-GT parameter alone was indicative of a lower overall survival (*p* = 0.038). In addition, the age (*p* < 0.001) was highly relevant to OS. In contrast, grading was not significantly relevant to OS (*p* = 0.412) in the univariable Cox regression (Table 6).

**Table 6:** Univariable Cox regression for OS.

**Variable**	**Coefficient (Odds)**	**95% CI**	***p***
MTV/chromogranin A	2.67	1.60–4.43	**<0.001**
γ-GT.log	1.41	1.02–1.95	**0.038**
LDH	1.00	0.99–1.01	0.615
Age	1.13	1.07–1.20	**<0.001**
Gender	0.78	0.34–1.76	0.549
Grading	1.45	0.60–3.50	0.412

Both MTV and/chromogranin A can be considered to represent the tumor burden. MTV, molecular tumor volume; γ-GT, gamma-glutamyl transferase; LDH, lactate dehydrogenase. Correlation of clinical factors on overall survival. Bold values are statistically significant.

In the multivariable Cox regression analysis, the combination of MTV and chromogranin A (HR 2.67; 95% CI 1.41–4.91; *p* = 0.002) was confirmed as a highly significant independent prognostic factor. In addition, LDH (HR 0.98; 95% CI 0.98–1.0; *p* = 0.007) and age (HR 1.15; 95% CI 1.08–1.23; p < 0.001) showed a significant impact on OS (Table 7). However, the re-analysis had only a minor effect on our result, so the changes were minimal (Supplementary Tables 1, 2). The highest impact was found in the covariate MTV/chromogranin A in our multivariable Cox regression for OS, which tended to be more significant after the exclusion of the four outliers.

**Table 7:** Multivariable Cox regression for OS.

**Covariate**	**Coefficient (Odds)**	**95% CI**	***p***
MTV/chromogranin A	2.67	1.46–4.91	**0.002**
γ-GT.log	0.97	0.60–1.55	0.890
LDH	0.99	0.98–1.00	**0.007**
Age	1.15	1.08–1.23	**<0.001**
Gender	0.75	0.28–1.99	0.560
Grading	1.26	0.46–3.47	0.653

Both MTV and/chromogranin A can be considered to represent the tumor burden. MTV, molecular tumor volume; γ-GT, gamma-glutamyl transferase; LDH, lactate dehydrogenase. Correlation of clinical factors on overall survival. Bold values are statistically significant.

**Figure 4:**
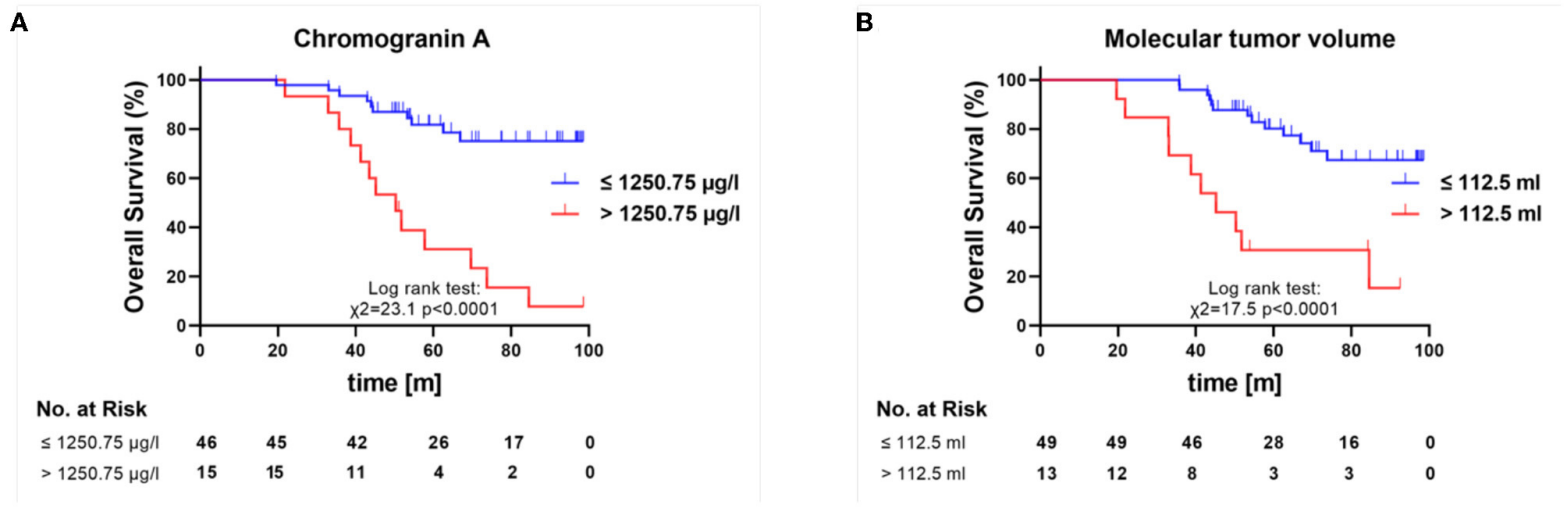
Kaplan–Meier overall survival curves for patients with high and low chromogranin A **(A)** and MTV **(B)** as based on ROC. m, months.

A ROC analysis was performed to analyze the 5-year survival rates and revealed the best cutoff value for the baseline MTV of >112.5 ml [sensitivity 91%; specificity 50%; AUC; 0.67 (95% CI 0.51–0.84, *p* = 0.043)] to identify patients with a worse 5-year survival rate. The best cutoff value for the baseline chromogranin A level was 1,250.75 μg/l [sensitivity 87%; specificity 56%; AUC 0.73 (95% CI 0.57–0.88, *p* = 0.0092)] to identify patients with a worse 5-year survival rate (Figure 4).

## Discussion

Following EMA approval in 2017, PRRT has emerged as a frequently used therapy for GEP-NETs G1 and G2.

NETTER1 was the first prospective phase 3 trial to demonstrate the benefit of PRRT. However, the authors of NETTER1 did not provide any information on prognostic factors. As stratification of treatment is of utmost interest, especially when very expensive therapies are used, we attempted to identify further prognostic markers.

In our study, we retrospectively evaluated prognostic factors prior to PRRT for the long-term outcome; the combination of MTV and chromogranin A was identified as a crucial surrogate marker for OS. In fact, MTV and chromogranin A represent the imaging-based and laboratory estimates of tumor burden, respectively. LDH was used since this marker is known to be a prognostic factor for different tumors (28). As 75% of NET patients are affected by liver metastases, γ-GT is a good biomarker for the effects of liver damage, and we selected and were able to confirm γ-GT as an additional indicator of treatment outcome in univariable Cox regression. In addition, our findings demonstrated that age plays a crucial role in terms of overall survival. Since the OS in NET was shown to be approximately 9 years (29), the long duration of the disease must be included in analyses of NET cohorts. However, out of all factors, the combination of MTV and chromogranin A showed the highest HR for OS.

At present, only two studies with more than 40 patients showed that the long-term survival of NET patients treated with PRRT was dependent on MTV. However, both studies did not report the potential confounding impact of age on OS in their analyses (30, 31). One study could not assess long-term prognostic markers due to a limited follow-up time of 31 months (30). Differences were found with regard to the cutoff values of MTV, which may be explained by the longer follow-up, the larger cohort, and the different segmentation methodologies in our study. Therefore, our patient cohort is one of the largest with a long follow-up period in the currently available literature, which demonstrates the impact of MTV on OS, and thus is the first study that evidenced MTV to be a risk factor independent of patients' age.

Since younger patients are known to recover faster and better from invasive interventions, such as surgery or other local therapies, and due to the known prolonged survival rates of NET patients, one might speculate that lowering the MTV prior to PRRT could improve the OS (32–34).

We also analyzed the change of the MTV after 2 cycles in comparison to the baseline PET/CT as described above. Non-responders to PRRT showed significantly worse overall survival. Therefore, in clinical practice, a PET/CT should be performed for therapy evaluation in patients after two treatment cycles, as non-responders may benefit from therapy adjustment.

Of course, these therapeutic adaptations have to be discussed in an interdisciplinary manner in consideration of all alternative treatment approaches.

In the NETTER-1 study, no statistically significant difference in the ultimate overall survival rate was shown between the PRRT arm and the control group, which might have been caused by a crossover of patients with PD in the SSA group (12).

Therefore, the results of the NETTER-1 study, including the limited OS, the low response rate of 15% together with a PFS of under 1 year, highlight the urgent need for prognostic factors and treatment monitoring measures to identify those patients for whom a change in treatment has to be considered.

The NETTER-1 study reported a 5-year OS rate of 35%, whereas, in our cohort, the 5-year OS rate was approximately 65%. These differences are most likely because not all patients in our cohort had previously a progression under SSA therapy. Patients with a high tumor burden or severe clinical complaints were sometimes directly treated with a combination of SSA therapy and PRRT.

NETTER-1 showed very similar rates of adverse events related to hematological disorders, reported to be approximately 3–4% grade 3/4 toxicities, as in our cohort. Moreover, in the prospective phase III study, as in our retrospective analysis, 1–2% developed MDS during the follow-up period.

## Limitations

A limitation of our study is the relatively small cohort size in a single center and the retrospective approach, as well as the fact that in the follow-up period, not all information about further therapies has been provided. The effectiveness of PRRT may be impacted by various primary tumor sites and different gradings. Moreover, due to the retrospective design and the patients' comorbidities, the clinical protocol is not uniform. Since many patients were treated prior to 2017, an 8-week therapy interval was not scheduled, which is common today. Furthermore, a different number of therapy cycles were performed in the patients. In addition, different SSTR targeting tracers, such as [^18^F]-SiFAlin-TATE, are available yet, which might show significantly different imaging properties from [^68^Ga]Ga-DOTA-TOC (35) or [^68^Ga]Ga-DOTA-TATE. In this respect, the cutoff values reported in this study cannot be used as an absolute reference, but our results indicate that the determination of other parameters, such as MTV, should also be used for therapy stratification in patients with NET.

## Conclusion

The limited long-term survival rates of the NETTER-1 study demonstrate the urgent need to find prognostic and follow-up markers. Our study demonstrated that a reference tissue-based MTV in combination with chromogranin A significantly affects long-term survival. Furthermore, the γ-GT showed a significant impact on OS in the univariable Cox regression. An interim PET/CT after two cycles can assist in identifying non-responders who might benefit from therapeutic adjustments. Further studies with larger sample sizes may be needed to better identify the optimal therapeutic sequence after progression to PRRT.

## Data availability statement

The raw data supporting the conclusions of this article will be made available by the authors, without undue reservation.

## Ethics statement

The study was approved by the IRB (Ethics Committee of the Faculty of Medicine of the Eberhard Karls University Tuebingen) of the University Hospital Tuebingen, and was conducted in accordance with the Declaration of Helsinki (Reference No. 530/220BO). The patients/participants provided their written informed consent to participate in this study.

## Author contributions

NT, HD, LZ, MH, and CF conceived and designed the study. NT, JS, JJ, GR, FF, HD, LZ, MH, and CF conducted the patient data, as well as the medical evaluation and analysis. NT, JS, JJ, GR, HD, and CF analyzed the data. NT prepared the figures. NT, JS, and CF wrote the first draft of the manuscript. LZ, HD, and MH contributed to the data interpretation and manuscript editing. All authors critically reviewed, read, and approved the final manuscript. All authors have read and agreed to the published version of the manuscript.
