# Supplementary figures and images for: Long-term prognostic factors for PRRT in neuroendocrine tumors
Source: Front Med (Lausanne). 2023 Jun 9;10:1169970. doi: 10.3389/fmed.2023.1169970 (PMC10288842; doi:10.3389/fmed.2023.1169970)

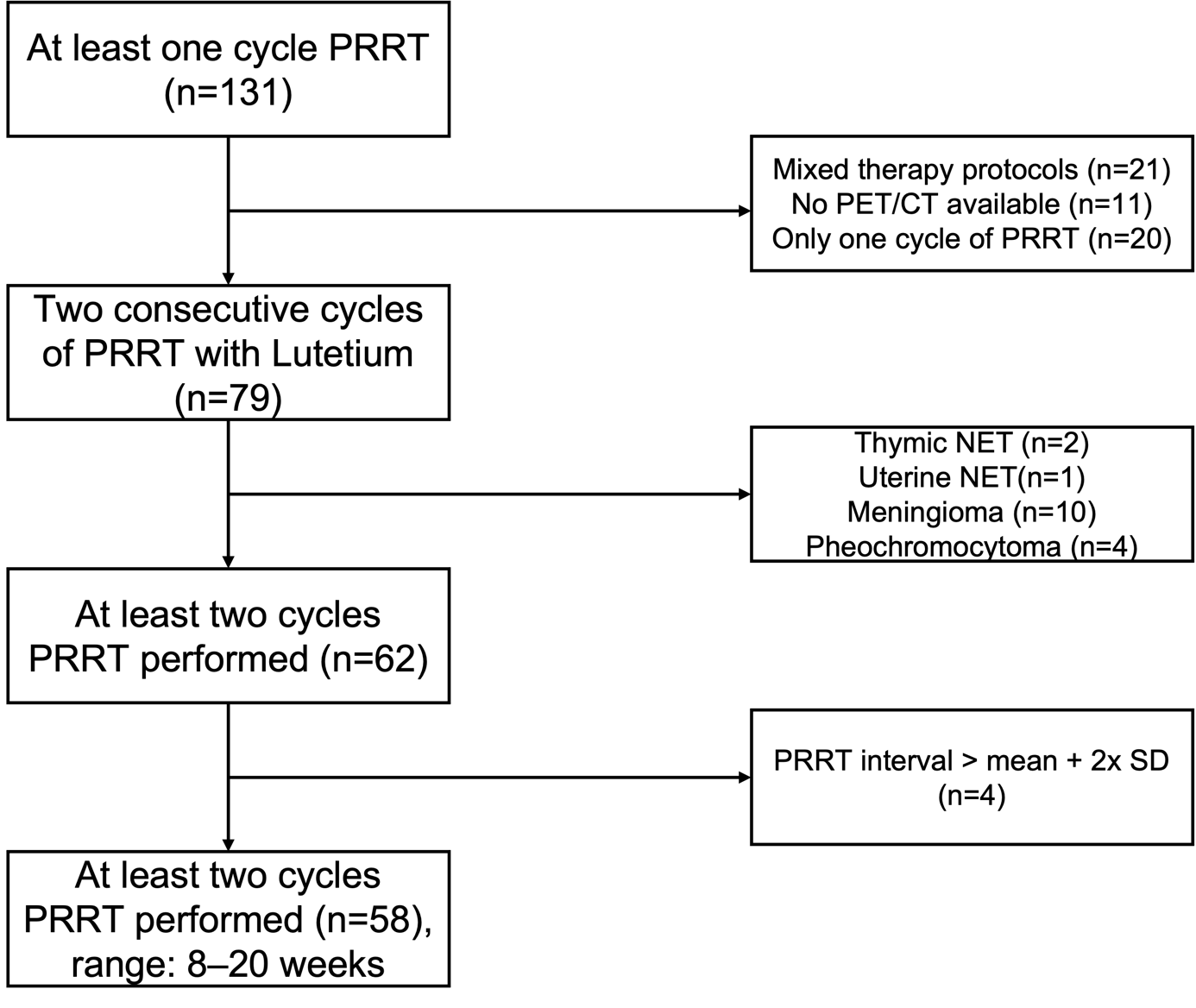

Supplement: Supplementary file 1 [file Image_1.tif]
