# Supplementary material for: Long-term prognostic factors for PRRT in neuroendocrine tumors
Source: Front Med (Lausanne). 2023 Jun 9;10:1169970. doi: 10.3389/fmed.2023.1169970 (PMC10288842; doi:10.3389/fmed.2023.1169970)
Supplement: Supplementary file 2 [file Table_1.docx]

**Supplementary Table 1.** *Univariable Cox-Regression for OS without outliner.*

| *Variable* | *Coefficient (Odds)* | *95% CI* | *p* | | | | |
| --- | --- | --- | --- | --- | --- | --- | --- |
| MTV/chromogranin A | 2.68 | 1.54-4.64 | **<0.001** |  |  |  |  |
| γ-GT.log | 1.40 | 0.94-1.36 | 0.058 |  |  |  |  |
| LDH | 1.00 | 0.99-1.01 | 0.799 |  |  |  |  |
| Age | 1.14 | 1.07-1.21 | **<0.001** |  |  |  |  |
| Gender | 0.76 | 0.32-1.82 | 0.540 |  |  |  |  |
| Grading | 1.49 | 0.57-3.85 | 0.413 |  |  |  |  |
|  |  |  |  |  |  |  |  |
