# Supplementary material for: Long-term prognostic factors for PRRT in neuroendocrine tumors
Source: Front Med (Lausanne). 2023 Jun 9;10:1169970. doi: 10.3389/fmed.2023.1169970 (PMC10288842; doi:10.3389/fmed.2023.1169970)
Supplement: Supplementary file 3 [file Table_2.docx]

**Supplementary Table 2.** *Multivariable Cox-Regression for OS without outliner.*

| \| *Covariate* \| *Coefficient (Odds)* \| *95% CI* \| *p* \| \| \| \| \| \| --- \| --- \| --- \| --- \| --- \| --- \| --- \| --- \| \| MTV/chromogranin A \| 5.59 \| 2.54 – 12.30 \| **<0.001** \| \| γ-GT.log \| 1.23 \| 0.77 – 1.97 \| 0.389 \| \| LDH \| 0.98 \| 0.96 – 0.99 \| **0.006** \| \| Age \| 1.28 \| 1.15 – 1.44 \| **<0.001** \| \| Gender \| 0.93 \| 0.33 – 2.62 \| 0.892 \| \| Grading \| 1.84 \| 0.61 – 5.51 \| 0.278 \|  \|  \|  \| |
| --- | --- | --- | --- | --- | --- | --- | --- | --- | --- | --- | --- | --- | --- | --- | --- | --- | --- | --- | --- | --- | --- | --- | --- | --- | --- | --- | --- | --- | --- | --- | --- | --- | --- | --- | --- |
